# Score‐based tests for parameter instability in ordinal factor models

**DOI:** 10.1111/bmsp.12392

**Published:** 2025-04-23

**Authors:** Franz Classe, Rudolf Debelak, Christoph Kern

**Affiliations:** ^1^ Deutsches Jugendinstitut e.V. Munich Germany; ^2^ Department of Psychology University of Zurich Zurich Switzerland; ^3^ Department of Statistics LMU Munich Munich Germany

**Keywords:** multidimensional item response theory, ordinal factor analysis, parameter instability, score test

## Abstract

We present a novel approach for computing model scores for ordinal factor models, that is, graded response models (GRMs) fitted with a limited information (LI) estimator. The method makes it possible to compute score‐based tests for parameter instability for ordinal factor models. This way, rapid execution of numerous parameter instability tests for multidimensional item response theory (MIRT) models is facilitated. We present a comparative analysis of the performance of the proposed score‐based tests for ordinal factor models in comparison to tests for GRMs fitted with a full information (FI) estimator. The new method has a good Type I error rate, high power and is computationally faster than FI estimation. We further illustrate that the proposed method works well with complex models in real data applications. The method is implemented in the *lavaan* package in R.

## 1 INTRODUCTION

Researchers investigating thought processes and cognitive abilities often use *item response theory* (IRT) models to measure multiple unobserved (or latent) variables like personality traits or proficiencies. One of the most widely applied IRT frameworks for observed variables with a small amount of ordered response categories is the *graded response model* (GRM; Samejima, 1969).

However, unidimensional IRT models, that is, models with only one latent variable, are often not able to model the full complexity of conceptually broad personality traits or abilities. Multidimensional item response theory (MIRT) models make it possible to analyse psychological assessment data such that underlying multidimensionality is captured (Reckase, 1997). The potential of such models for large‐scale test and questionnaire evaluation and development has been emphasized numerous times (Bean & Bowen, 2021; Immekus et al., 2019; ten Holt et al., 2010). A major advantage of MIRT models is their flexibility, because latent covariance structures, hierarchical latent variable structures or within‐item multidimensionality can be included in the model (Hartig & Höhler, 2009). In this paper, we develop an approach to compute model scores for a special kind of (multidimensional) IRT model, namely the ordinal factor model. This opens up novel avenues in latent variable modelling.

A popular estimation method in IRT is *marginal maximum likelihood* (MML) estimation via the *expectation maximization* (EM) algorithm (Bock & Aitkin, 1981; Jöreskog & Moustaki, 2006). This approach is commonly considered a full information (FI) estimation method because all distinct values on the observed variables are used (Bolt, 2005). However, parameter estimation for MIRT models via this FI method is computationally demanding, especially if there is more than one dimension (i.e. latent variable) (Forero & Maydeu‐Olivares, 2009; Muraki & Carlson, 1995) as the complexity of the EM algorithm increases exponentially with the number of latent variables. In contrast, the complexity of the *Metropolis‐Hastings Robbins‐Monro* (MH‐RM) increases linearly with the number of latent variables. It has proven to be accurate and relatively efficient for MIRT model estimation (Cai, 2010; Yavuz & Hambleton, 2017). However, compared to alternative approaches, model estimation with the MH‐RM algorithm is still computationally demanding if more than one latent variable is specified in the model.

According to Liu et al. (2018), contemporary MIRT is a convergence of developments from test theory and confirmatory factor analysis (CFA). This means that certain types of CFA models and IRT models are equivalent (Takane & De Leeuw, 1987). Building on this assumption, Muthén (1984) proposed a *limited information*(LI) approach in which the polychoric correlation matrix of the response variables is used for parameter estimation. LI methods are usually computationally more efficient than FI methods and commonly used in practice. *Pairwise maximum likelihood* (PML) is a specific type of LI method which (like MML) uses a likelihood function for parameter estimation and maximizes the log‐likelihoods associated with all item pairs (Katsikatsou et al., 2012). In this article, however, we focus on the most widely used LI estimate, which goes back to Muthén (1984). In the following, we therefore use ordinal factor analysis as a broad term for (multidimensional) IRT models estimated via polychorics (Maydeu‐Olivares et al., 2011; Shi et al., 2020).

In IRT, it is generally assumed that the item parameters are independent of any covariates of the observed variables in the population of test takers. Such covariates may be demographic characteristics such as age, gender or education level. Violations of this assumption are interpreted as differential item functioning (DIF; Millsap, 2012; Osterlind & Everson, 2009). In practice, DIF may be detected by pre‐specifying subgroups for which measurement invariance is not assumed. Alternatively, one can use the score‐based test for parameter instability (Zeileis & Hornik, 2007) to detect DIF. This test focuses on identifying parameter instability through an analysis of the relation between model parameters and person covariates. It tests the null hypothesis that model parameters remain invariant across all values of person covariates. The score‐based test is computed using the model scores, that is, the partial derivative of the casewise contributions to the objective function with respect to the model parameters (Merkle & Zeileis, 2013). It has been applied to a variety of different psychometric models, including factor analysis (Merkle et al., 2014), Bradley–Terry models (Strobl et al., 2011), binary and polytomous Rasch models (Komboz et al., 2018; Strobl et al., 2015), logistic IRT models (Debelak & Strobl, 2019a), mixed models (Fokkema et al., 2018), as well as the two‐parameter normal ogive model via the PML estimation method (Wang, Strobl, et al., 2018). It is, however, currently not applicable to the GRM via ordinal factor analysis (i.e. LI estimation via polychorics).

We propose a method to efficiently approximate individual model scores, that is, the partial derivative of the casewise contributions to the objective function, for ordinal factor models. With this method, it is possible to apply score‐based tests to such models. The score‐based test for parameter instability can therefore be applied to MIRT models, specifically multidimensional GRMs, with reasonable computational effort.

We simulate data based on two (uni‐ and multidimensional) GRMs and systematically investigate the performance of the proposed score‐based test. We compare our approach to tests based on models fitted with FI estimation under various conditions. Furthermore, we investigate the distribution of the scores estimated with the proposed method by comparing the correlations of model score contributions from different fitting approaches.

In the following, we describe the methodological background of this paper and how score‐based tests for parameter instability can be used to detect DIF. We further introduce ordinal factor analysis and subsequently present our approach to compute individual model scores for ordinal factor models. Next, we present simulations with different scenarios to test the performance of score‐based tests based on models fitted with both LI and FI estimation. Following this, we apply models fitted via different estimation methods to real data and compare the computation times and the results of the score‐based tests for parameter instability. In the last section, the results are discussed.

## 2 METHODOLOGICAL BACKGROUND

### 2.1 Model definition

In IRT models, the latent variable, denoted as ξ, typically represents the respondent's ability that is assumed to underlie their response patterns. Let the graded responses be represented by the observed variable $Y_{j}$, for a given item j. Usually, IRT models are estimated based on ordered observed variables, wherein i=1,…,n, respondents choose from a range of ordered response categories $k_{j}=1,\text{…},l_{j},$ for items j=1,…,p. For simplicity, we assume that all items have the same number of categories, such that $k_{j}=k\forall j=1,\text{…},p$. In a multidimensional GRM, ξ is a m×1 vector containing all latent variables $\xi_{q}\forall q=1,\text{…},m$. An observed variable $Y_{j}$ may be associated with multiple latent variables.

In the GRM, the probability of answering in a category smaller or equal to a certain ordered category k depends on the (multidimensional) distribution of the latent variables as well as on the model's parameters. The threshold parameters $\tau_{jk}$ represent the boundaries between the categories. The threshold locations determine the difficulties of the item categories. The discrimination parameters $\lambda_{j}$ denote the loadings of the items on the latent variables. The relationship between the latent variable and the response variables is defined by the cumulative category response function, that is

(1) $$P(Y_{j}\leq k|\xi ,\theta )=\Phi (\tau_{jk}-\lambda_{j}^{\prime }\xi ),$$

where Φ is the distribution function of the standard normal distribution. It is used as a link function to convert a linear function into a probability function. The link function is also known as probit function or normal ogive function. Alternatively, a logit function can be used for the GRM (Samejima, 1997).

The model parameter vector θ contains all freely estimated threshold parameters $\tau_{jk}$, all freely estimated discrimination parameters $\lambda_{qj}$ that make up the m×1 vector $\lambda_{j}$, as well as all freely estimated latent variable variances and covariances, such that

(2) $$\begin{array}{cc} \theta & =\{\tau_{11},\text{…},\tau_{pl},\lambda_{11},\text{…},\lambda_{mp},\\ & \mathrm{Var}(\xi_{1}),\text{…},\mathrm{Var}(\xi_{m}),\\ & \text{Cov}(\xi_{1},\xi_{2}),\text{…},\text{Cov}(\xi_{m-1},\xi_{m})\}. \end{array}$$

Note that $\mathrm{Var}(\xi_{q})$ is fixed to 1 if $\lambda_{q1}$ is freely estimated (and vice versa).

### 2.2 Differential item functioning

In the context of IRT models, differential item functioning (DIF) arises when an item's characteristics are related to person covariates. For instance, covariates such as ethnicity, education or gender may have an impact on, for example, the difficulty of an item. This means that one or more items of a test have different difficulties for subgroups with different ethnicity, education or gender. Let Z be a covariate that induces such a DIF effect. In this case, the item parameters in θ deviate across the distribution of Z. If Z is independent of the latent variable, DIF occurs when the probability of responding to an item in a particular category differs between two individuals with the same ability (i.e. the same values on ξ) solely due to their different values on Z. Practically, undetected DIF may lead to a misinterpretation of group differences concerning latent variables (Wang, Strobl, et al., 2018). Thus, DIF analyses are important in the practice of test validation (Walker, 2011). Note, however, that DIF is fundamentally different to *impact*, which means that the distribution of the latent variable depends on Z. For example, two subgroups with different ethnicity, education and gender may differ with respect to the values on the latent variable, but the difficulties of the test items may be equal across these groups. If impact of the latent variable is expected, testing for DIF requires a model in which the item parameters can differ between groups while controlling for group differences in the latent variable distribution (Belzak & Bauer, 2020; Sterner et al., 2024).

As mentioned above, DIF is closely related to the concept of measurement invariance, which is a concept primarily used in factor analysis. Measurement invariance in a model is established by the conditional independence of all observed variables and all potentially confounding covariates (Sterner et al., 2024). For a model with p observed response variables, this rule can be expressed as

(3) $$Y_{i}⫫Z|\xi_{i},\forall i=1,\text{…},p,$$

where $Y_{i}$ is the observed response variable for item i, Z is the vector of all potentially confounding covariates and $\xi_{i}$ is the vector of latent variables pertaining to item i. For an MIRT model, it follows from Equation (3) that the $\xi_{i}$‐conditional probability of answering to item i is independent from Z, which means that there is no DIF. For simplicity, we refer to DIF as measurement non‐invariance in IRT models.

Traditional approaches of empirical testing for DIF require the prespecification of subgroups for which DIF is assumed. For a focal subgroup and a reference subgroup, differences in item parameters can be tested for. This can be done for single items. This way, one can detect items with DIF so that this item can, for instance, be removed from the scale. For example, the subgroups tested for DIF are divided at the median of the metric covariate Z. In this case, two distinct subgroups are defined and the likelihood ratio (LR) test can be applied. With the LR test, an augmented model, permitting variation in all item parameters across the two groups, is tested against a baseline model where all item parameters are constrained to be equal between the reference and focal groups (Bulut & Suh, 2017). If the likelihood ratio of these two models is significantly different from one, researchers must assume the presence of DIF between these two groups. In practice, prior specification of subgroups potentially subjected to DIF can be difficult, especially in situations where there are a multitude of potential splitting points on Z. As researchers might not have strong assumptions which groups might be affected by DIF, certain subgroups exhibiting DIF might remain undiscovered.

### 2.3 Score‐based test for parameter instability

A solution to this problem was proposed by Zeileis and Hornik (2007) who presented a family of generalized M‐fluctuation tests for testing parameter instability with respect to observed metric, ordinal and categorical variables. In the following, we refer to these tests as *score‐based tests*. They are applicable to a wide range of IRT models to detect DIF (Debelak & Strobl, 2019a; Schneider et al., 2022). The score‐based test is a global test for parameter instability. Usually, all freely estimated model parameters are tested for instability when the score‐based test is applied to a fitted model. The application of the score‐based test to MIRT models for DIF detection presupposes that no impact of the latent variable is assumed. If differences in the latent variable are assumed across prespecified groups, one can apply the score‐based test to a multiple‐group MIRT model, in which the means and variances of the latent variable can differ for predefined subgroups (Bock & Zimowski, 1997; Debelak et al., 2022; Debelak & Strobl, 2019a). Note that such multiple‐group models require one or more anchor items to make sure that the latent variable is measured on the same scale across groups. In single‐group MIRT models, such group differences in the latent variable distributions are mistaken for DIF if the score‐based test is used for DIF detection. In this paper, we only consider single‐group MIRT models without differences in the latent variable between subgroups.

Another prerequisite for the score‐based test is that an *M‐estimator* is used to fit the model. If this is the case, parameter instability of the fitted model with respect to a covariate can be investigated. Following Stefanski and Boos (2002), an M‐estimator $\hat{\theta }$ is defined as the solution to the equation

(4) $$\overset{n}{\underset{i=1}{\sum }}\psi (y_{i},\hat{\theta })=0,$$

where ψ is a 1×‖θ‖ matrix. Note that ‖·‖ denotes vector length.

The function ψ is the first derivative of the objective function that is minimized to estimate the model parameters. In the context of marginal maximum likelihood (MML) estimation, which is a common full information estimation approach for IRT models, the objective function is the negative log‐likelihood function. Following Baker and Kim (2004, pp. 160–164), the marginal likelihood L of the observed data is

(5) $$L(Y,\theta )=\overset{n}{\underset{i=1}{\prod }}P(y_{i}),$$

where $y_{i}=\{y_{i1},\text{…},y_{im}\}$ is the response pattern of respondent i. The probability of the individual response pattern of respondent i is

(6) $$P(y_{i})=\int P(y_{i}|\xi_{i},\theta )g(\xi_{i})d\xi_{i}$$

where $\xi_{i}$ are the values of respondent i on the latent variables (in IRT these are also referred to as person parameters). These values are drawn from the specific multivariate distribution $g(\xi_{i})$. Under the usual conditional independence assumption of the GRM, $P(y_{i}|\xi_{i},\theta )$ follows from Equation (1). The derivative of the log likelihood with respect to some parameter x is

(7) $$\frac{\partial \log L(Y,x)}{\partial x}=\overset{n}{\underset{i=1}{\sum }}\frac{1}{P(y_{i})}\frac{\partial }{\partial x}P(y_{i})=\overset{n}{\underset{i=1}{\sum }}\psi (y_{i},x)=0,$$

where $\frac{\partial P(y_{i})}{\partial x}$ differs for each parameter x in θ.^1^ The individual contributions to the first derivative of the log likelihood with respect to the M‐estimator $\hat{\theta }$ are also referred to as the *score contributions* of the fitted model. This is why the generalized M‐fluctuation test is called score‐based test.

The null hypothesis of the score‐based test, which states that model parameters are invariant, is rejected if the empirical fluctuation during parameter estimation with respect to Z is improbably large. To estimate the empirical fluctuation, the individual model scores $\psi (y_{i},\hat{\theta })$ are computed for all individuals i in the sample. If the model parameters deviate across the distribution of a metric covariate Z, then a transition from positive to negative scores for lower values on Z to higher values on Z (or vice versa) is expected (see left hand side of Figure A3). The scores are then cumulated according to the order of the covariate of interest Z to compute the cumulative score process

(8) $$\text{CSP}(H)={\hat{B}}^{-1/2}\frac{1}{\sqrt{n}}\overset{H}{\underset{h=1}{\sum }}\psi (y_{\left(h|Z\right)},\hat{\theta }),$$

where (h|Z) denotes the h‐th ordered observation with respect to the covariate Z. The transition from positive to negative scores is captured as a clearly noticeable peak in the cumulative sum process (see right hand side of Figure A3). The sum process is scaled by an estimate $\hat{B}$ for the covariance matrix $\text{cov}(\psi (Y,\hat{\theta }))$ to decorrelate the scores so that the score processes for all parameter estimates in $\hat{\theta }$ are independent from each other. By analysing the CSP, a possible systematic change from positive to negative scores across the covariate can be detected.

Different kinds of test statistics can be derived from the CSP to capture the fluctuation across all parameter estimates in $\hat{\theta }$. For metric covariates, the maximum Lagrange multiplier (*maxLM*), the double maximum (*DM*) and the Cramér‐von‐Mises (*CvM*) test statistics are available. The unordered LM test statistic (*LMuo*), which is based on the sum of the values in each category, is used to assess instability in relation to categorical covariates where it is not possible to order the values (Merkle & Zeileis, 2013). For ordered covariates, the ordered maximum LM (*maxLMo*) and the “weighted” double maximum (*WDMo*) statistic can be used (Merkle et al., 2014). Critical values associated with these test statistics can either be obtained through closed‐form solutions of certain functions (*DM*, *WDMo*, *LMuo*), through tables of critical values obtained from simulation (*maxLM*, *CvM*) or through repeated simulation of Brownian Bridges (*maxLMo*). All these test statistics are implemented in the strucchange package in R (Zeileis et al., 2015).

As mentioned before, the score‐based test for parameter instability is applicable for many different kinds of IRT models. However, MIRT models are commonly fitted via FI estimation, namely with the MML estimator (Schneider et al., 2022), such that individual score contributions can be computed as terms of the derivative of the marginal log‐likelihood (Baker & Kim, 2004; Debelak & Strobl, 2019a). For simple IRT models, such as the Rasch model (Rasch, 1960) or the 2PL model by Birnbaum (1968), FI estimation is very efficient and repeated model fittings in a recursive partitioning algorithm are computationally feasible (see Komboz et al., 2018; Strobl et al., 2015). However, this is not the case for complex MIRT models. For these models, LI estimation, as common in ordinal factor analysis, is much quicker (Forero & Maydeu‐Olivares, 2009). Therefore, a method for estimating individual score contributions for ordinal factor models is an important prerequisite for the efficient application of the score‐based test.

### 2.4 Full information estimation

The marginal maximum likelihood (MML) estimation approach via the expectation maximization (EM) algorithm (Bock & Aitkin, 1981; Jöreskog & Moustaki, 2006) iteratively estimates the true probabilities of each observed response pattern. In the first step of the algorithm, the latent variable is estimated (E‐step), and in the second step, the model parameters are optimized (M‐step). However, for this full information (FI) estimation method, multidimensional integrals are evaluated in the estimation process. Intensive computations are required, especially if latent variables in the MIRT model are correlated (Forero & Maydeu‐Olivares, 2009). Efforts to reduce computation time have been made by Meng and Schilling (1996) via the Monte Carlo EM algorithm and later via the Markov Chain Monte Carlo (MCMC) algorithm (Bolt & Lall, 2003; Kim & Bolt, 2007). The Metropolis‐Hastings Robbins‐Monro (MH‐RM) algorithm is building on these advances (Cai, 2010). The algorithm has initially been proposed for exploratory factor analysis. It synthesizes a type of MCMC algorithm, the Metropolis–Hastings algorithm (Hastings, 1970; Metropolis et al., 1953), with the Robbins–Monro method (Robbins & Monro, 1951) for stochastic approximation. Its complexity increases linearly with the number of latent variables. In the following, we will compare the performance of the MML estimation approach via the MH‐RM algorithm with the performance of the limited information estimation approach used for ordinal factor analysis. We will focus on computation time and score‐based parameter instability test results.

### 2.5 Ordinal factor analysis

Using the classic maximum likelihood approach for CFA (see Jöreskog, 1969) to fit (multidimensional) IRT models introduces model misspecification because the common CFA assumes linear relationships between continuous and normally distributed observed variables and continuous factors (Maydeu‐Olivares et al., 2011). Thus, in order to include ordered observed variables in CFA models, continuous latent response variables $Y^{*}$ are assumed to underlie the observed ordered variables Y (Takane & De Leeuw, 1987), that is

(9) $$Y^{*}=\lambda^{\prime }\xi +\epsilon .$$

The mean vector of $Y^{*}$ is $E(Y^{*})=\lambda \alpha ,$ where α is the mean vector of ξ. The (co‐)variances matrix of the $Y^{*}$ is $\mathrm{Var}(Y^{*})=\lambda^{\prime }\Psi \lambda +\Theta$, where Ψ is the covariance matrix of ξ and Θ is the covariance matrix of ϵ. The scale of the latent response variables is a priori indeterminate. Thus, in multi‐group models, the latent response variables are usually standardized for at least one group (Muthén, 1984).

The latent response variable of item j is related to the observed ordered variable of item j via a threshold relation, that is

(10) $$Y_{j}=k\mathrm{if}\tau_{j(k-1)}<y_{j}^{*}\leq \tau_{jk},$$

where $\tau_{j0}=-\infty$ and $\tau_{jl}=\infty$. Thus, for every item j, there is one threshold parameter $\tau_{jk}$ less than the total number of ordered categories l within item j. Note that the probability of $Y_{j}$ being greater than k may be derived from the threshold parameters, that is,

(11) $$P(Y_{j}>k)=P(Y_{j}^{*}>\tau_{jk})=\Phi (-\tau_{jk}).$$

Building on this assumption, Muthén (1984) proposed a method in which parameters of CFA models including ordered observed variables are estimated by minimizing the discrepancy between the polychoric correlation matrix of the observed variables and the model‐implied covariance matrix. Parameter estimation via polychorics is also referred to as a form of limited information (LI) estimation, as it only uses information from bivariate relations of the observed variables. The estimation of the thresholds, as defined in Equation (10), is performed as a first step in the model fitting process. Furthermore, in this phase, bivariate polychoric correlations $\rho_{js}$ are computed for all j,s=1,…,p when j≠s, following the approach established by Olsson (1979). These polychoric correlations quantify the degree of linear dependence between the variables $Y_{j}^{*}$ and $Y_{s}^{*}$ for j≠s.

After the estimation of thresholds and polychoric correlations, the model parameters in θ are estimated through minimization of the objective function

(12) $$F_{\mathrm{OFA}}(\theta )={\left[\hat{\kappa }-\kappa (\theta )\right]}^{\prime }W^{-1}[\hat{\kappa }-\kappa (\theta )],$$

where $\hat{\kappa }$ and κ(θ) are the vectors of the sample and model implied polychoric correlation matrices. Different choices for the positive‐definite weight matrix W lead to different estimators (Shi et al., 2020). In Weighted Least Squares (WLS; Muthén, 1984) estimation, W is the asymptotic covariance matrix of $\hat{\kappa }$. The WLS estimator may produce unstable results for small sample sizes and large models (Flora & Curran, 2004; Garnier‐Villarreal et al., 2021; Wang, Su, et al., 2018). However, it usually performs equally well or better than FI estimation for large sample sizes (Forero & Maydeu‐Olivares, 2009). In this paper, we therefore focus on the WLS estimator for ordinal factor analysis.

### 2.6 Approximated scores for ordinal factor analysis

As we assume a specific model structure for a multidimensional GRM, we may denote the assumed model (see Equation 1) as the *structured* model, or $H_{0}$. It may be tested against the *unstructured* or *saturated* model ($H_{1}$) that does not impose any restrictions on the thresholds or the covariance matrix. The vector κ contains the saturated model parameters ${\left(\tau ,\sigma^{*}\right)}^{\prime }$, where τ is the vector of threshold parameters, and $\sigma^{*}=\text{vech}[\text{Cov}(Y^{*})]$ contains the vectorized non‐redundant elements of the model implied covariance matrix. The size of κ is [p(l−1)+p(p−1)/2]×1 which we refer to as $p^{*}\times 1$ in the following.

The first derivative of κ with respect to θ is

(13) $$\Delta =\frac{\partial \kappa (\theta )}{\partial \theta }=\left(\begin{array}{c} \frac{\partial \tau (\theta )}{\partial \theta }\\ \frac{\partial \sigma^{*}(\theta )}{\partial \theta } \end{array}\right).$$

We apply the chain rule to get the first derivative of the objective function with respect to θ, that is the 1×‖θ‖ matrix

(14) $$\frac{\partial F_{\mathrm{OFA}}(\theta )}{\partial \theta }=\frac{\partial F_{\mathrm{OFA}}(\kappa )}{\partial \kappa }\frac{\partial \kappa (\theta )}{\partial \theta }=-2{\left[\hat{\kappa }-\kappa (\theta )\right]}^{\prime }W^{-1}\Delta .$$

Note that Equation (14) is not an individual function, meaning that it does not refer to a single observation i and cannot be used for the score‐based parameter instability test. To our knowledge, it is not possible, with reasonable effort, to formulate the gradient of Equation (12) as an individual function.

Therefore, to compute scores that can then be used for the score‐based parameter instability test, we focus on an alternative approach to MML estimation. Muthén (1997) and Reboussin and Liang (1998) proposed a *generalized estimating equations* (GEE) approach for the estimation of parameters in (multidimensional) latent variable models with ordered response variables. In Appendix S1, we describe how the GEE estimation method is applied to MIRT models based on non‐binary response variables. This approach minimizes a set of estimating equations, that is

(15) $$\overset{n}{\underset{i=1}{\sum }}\Delta^{\prime }W_{\mathrm{GEE}}^{-1}e_{i}=0,$$

where $e_{i}$ is the vector of empirical deviations of the first‐ and second‐order empirical moments in the data from the true first‐ and second‐order moments (see Equations 10–14 in Appendix S1). The first‐order empirical moments in the data are the indicator variables, that is,

(16) $$1_{y_{i}>k}=\left\{\begin{array}{cc} 1,\; & \text{if}\;y_{i}>k\\ 0,\; & \text{otherwise}, \end{array}\right.$$

for all individuals i=1,…,n, all items j=1,…,p, and all categories minus one k=1,…,(l−1). The weight matrix used in GEE estimation, that is, $W_{\mathrm{GEE}}$, is defined as the working covariance matrix of first‐ and second‐order empirical moments of individual i (see Equations 16–18 in Appendix S1). The Δ‐matrix is the derivative of the saturated model with respect to the model parameters θ (see Equation 19 in Appendix S1).

In contrast to Equation (14), the estimating equations in Equation (15) are individual functions that each refer to a single observation i and add up to zero. They are the individual contributions to the derivative of the objective function of the GEE approach. The model parameters in θ are estimated by iteratively updating the estimator, that is, solving the set of quadratic estimating equations for θ. The solution to the set of quadratic estimating equations are the model scores obtained through GEE estimation. Using the GEE estimation approach leads to slightly different parameter estimates than ordinal factor analysis (i.e. WLS). Our goal is to approximate the GEE scores that would have resulted if the parameters estimated using the GEE approach were exactly the same as those estimated using ordinal factor analysis. We claim that these approximated scores can be used for the score‐based parameter instability test.

We learn from GEE estimation (e.g. equation 28 in Muthén, 1997) that an empirical deviation vector $e_{i}$, defined on the individual level, can be used for the individual estimating function (Equation 15). Let an alternative set of individual estimating equations be

(17) $$\begin{array}{ll} & {s^{*}}_{i}=\left(\begin{array}{c} \left(y_{i1}^{*}-\tau_{1})(y_{i2}^{*}-\tau_{2}\right)\\ \left(y_{i1}^{*}-\tau_{1})(y_{i3}^{*}-\tau_{3}\right)\\ \vdots \\ \left(y_{ip-1}^{*}-\tau_{p-1})(y_{ip}^{*}-\tau_{p}\right) \end{array}\right),\\ & \;\overset{n}{\underset{i=1}{\sum }}\Delta^{\prime }W^{-1}\left(\begin{array}{c} {y^{*}}_{i}-\tau \\ {s^{*}}_{i}-\sigma^{*} \end{array}\right)=0,\; \end{array}$$

where ${y^{*}}_{i}$ contains the values of individual i on the latent response variables for all items j=1,…,p. The vector ${s^{*}}_{i}$ can be referred to as the vector of the true second‐order moments. Note that both ${y^{*}}_{i}$ and ${s^{*}}_{i}$ cannot be observed. However, for a direct translation of the GEE estimation method to ordinal factor analysis, it would be necessary to observe ${y^{*}}_{i}$ and ${s^{*}}_{i}$. Also, for such a translation, the $p^{*}\times p^{*}$ matrix W in Equation (17) would be an estimator of the working covariance matrix of the vectors ${\left({y^{*}}_{i},{s^{*}}_{i}\right)}^{\prime }$ across all individuals i=1,…,n. In the following, we will show how to approximate the GEE scores without having to observe ${y^{*}}_{i}$ and ${s^{*}}_{i}$.

Let us assume that the latent response variables in the model be normally distributed and that the model's residuals $\epsilon_{j}=Y_{j}^{*}-\lambda_{j}^{\prime }\xi$ (see Equation 9) are independent and identically distributed. If this is the case, then $\hat{\kappa }=\kappa (\theta )$, that is, the assumed model fits the data perfectly and the empirical deviation vector in Equation (17) is equal to $\left(\begin{array}{c} {y^{*}}_{i}-{\bar{y}}^{*}\\ {s^{*}}_{i}-{\bar{s}}^{*} \end{array}\right)$, where $\bar{\cdot }$ represents the arithmetic mean.

To compute individual score contributions based on Equation (12), we apply the logic of Equation (17) to the non‐binary case. The aim is to mimic the scores produced by the estimation function in Equation (17). However, the individual values of the latent response variable distribution $y^{*}$ are not identifiable. Thus, the true second‐order moments $s^{*}$ are not identifiable either. We therefore replace the empirical deviation vector with $\left(\begin{array}{c} \text{vec}(1_{y_{i}})-\text{vec}(\bar{1_{Y}})\\ {\text{s}}_{i}-\bar{s} \end{array}\right)$. The vector $\text{vec}(1_{y_{i}})$ contains the indicator variables for all items j=1,…,p, and all categories minus one k=1,…,(l−1) (see Equation 10 in Appendix S1). The vector $\text{vec}(\bar{1_{Y}})$ of size p(l−1) contains the arithmetic means of the indicator variables across all individuals. Furthermore, we replace the weight matrix of Equation (17) with the weight matrix of Equation (12). This way, we account for the multivariate non‐normality within the observed variable distribution. Thus, we claim that the individual score contributions of an ordinal factor model fitted using WLS can be estimated as follows

(18) $$\begin{array}{ll} s_{i} & =\left(\begin{array}{c} \left(y_{i1}-{\bar{y}}_{1})(y_{i2}-{\bar{y}}_{2}\right)\\ \left(y_{i1}-{\bar{y}}_{1})(y_{i3}-{\bar{y}}_{3}\right)\\ \vdots \\ \left(y_{ip-1}-{\bar{y}}_{p-1})(y_{ip}-{\bar{y}}_{p}\right) \end{array}\right),\\ \overset{n}{\underset{i=1}{\sum }}\tilde{\psi }(y_{i},\theta ) & =\overset{n}{\underset{i=1}{\sum }}\Delta^{\prime }W^{-1}\left(\begin{array}{c} \text{vec}(1_{y_{i}})-\text{vec}(\bar{1_{Y}})\\ {\text{s}}_{i}-\bar{s} \end{array}\right)=0.\; \end{array}$$

We refer to Equation (18) as the *approximated score function* of the WLS estimation method that can be used for the score‐based parameter instability test.

### 2.7 Computational details

The R implementation of the proposed method, replication materials for all simulations, all simulation results as well as Appendix S1 are provided in the following OSF repository: https://osf.io/hmwpc/?view{_}only=69ed2919e7a64db2b0354f99243c307c. All simulations and real data applications were executed on a 20 core, 170 GB RAM server. The proposed method to compute individual model scores for ordinal factor models is implemented in the functions lavScores() and estfun.lavaan() in the latest version (since version 0.6‐18) of lavaan (Rosseel, 2012).

## 3 SIMULATION

**FIGURE A1:**
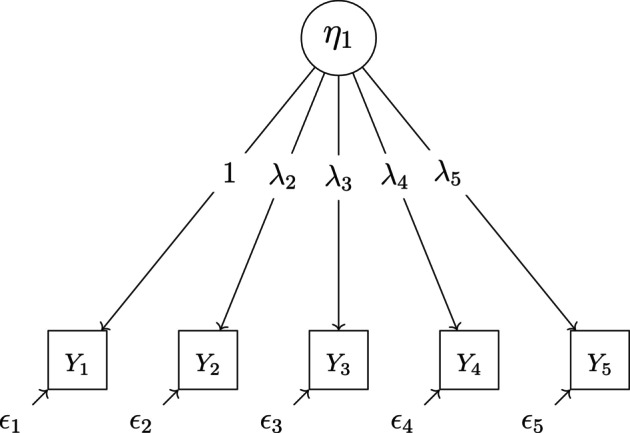
Unidimensional graded response model (GRM) with five items.

**FIGURE A2:**
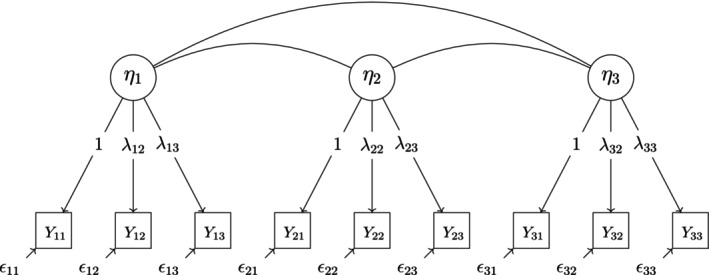
Multidimensional graded response model (GRM) with three non‐orthogonal latent variables and nine items.

**FIGURE A3:**
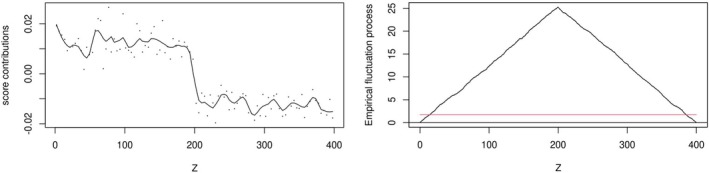
Score and CSP distribution (illustration inspired by figure 2 in Strobl et al., 2015).

We simulated data to fit two different IRT models: a unidimensional model with five observed variables $Y_{j}$ (Figure A1) and a multidimensional model with nine observed variables $Y_{j}$ (Figure A2). To simulate model‐compliant data, first, true latent variable scores were simulated for all latent variables in the model. Then, values of the conditional probabilities $P(Y_{j}=k|\xi ,\theta )$ were computed for all categories of all items. On the basis of these conditional probabilities, values for five ordinal response variables with k categories each were sampled.

From these conditional probability functions, DIF effect sizes can be calculated. Following Chalmers (2023), the scoring function that is

(19) $$S(\xi ,\theta )=\overset{l}{\underset{k=1}{\sum }}(k-1)\cdot P(Y_{j}=k|\xi ,\theta ),$$

is used to compute the DIF effect size of an item j. The *Noncompensoratory DIF* (NCDIF) value quantifies the average deviation of the response function of an item j between a focal group (F) and a reference group (R). It is defined as

(20) $$\text{NCDIF}=\frac{\sum_{i=1}^{n_{F}}{\left[S(\xi_{i},\theta_{F})-S(\xi_{i},\theta_{R})\right]}^{2}}{n_{F}}.$$

Using the true values for $\xi_{i}$, $\theta_{F}$ and $\theta_{R}$ from the simulation, we are able to compute the true NCDIF values of the items in the simulated data sets. To illustrate how parameter fluctuation affects parameter estimation, we report a DIF effect size, that is, the NCDIF value, for one specific item (Item 2) in 24 different simulation scenarios: two different models, that is, the unidimensional model (Figure A1) and the multidimensional model (Figure A2), four different numbers of threshold parameters k∈{1,2,4,6} and three different scenarios for parameter fluctuation in the data (see below). For each scenario, we simulate 1000 simulation samples of n=1000 in which there is a focal group and a reference group. For each group, the parameters of the (multidimensional) GRM are randomly drawn. For each sample, the NCDIF values are computed. The average NCDIF values (i.e. the arithmetric means) are shown in Table A3.

To test the performance of the score‐based test, we created 36 different simulation scenarios for each model: four different numbers of threshold parameters k∈{1,2,4,6}, which means that the simulated ordinal observed variables $Y_{j}$ have two, three, five or seven categories, three different sample sizes n∈{500,1000,2000}, and three different scenarios for parameter fluctuation in the data. For each of the simulated samples, we created one numerical covariate ($Z_{\mathrm{num}}$) ranging from 1 to 200, one ordinal ($Z_{\mathrm{ord}}$) and one categorical ($Z_{\mathrm{cat}}$) covariate with scores on a five‐point response scale. Each simulation sample consists of a focal and a reference group of size n/2 that both fit the corresponding model but have different parameter values.

The three simulated scenarios for parameter fluctuation are: All parameter values differ between the focal and the reference group, only the threshold parameters of the first item $\tau_{1k}$ (for the unidimensional model) or the threshold parameters of the first and the second item (for the multidimensional model) differ between the focal and the reference groups, or only the discrimination parameters $\lambda_{j}$ (of all items) differ between the two subsets. Thus, each simulation sample for each model for each simulation scenario is of size n and exhibits DIF with respect to the covariates $Z_{\mathrm{num}}$, $Z_{\mathrm{ord}}$ and $Z_{\mathrm{cat}}$. This means that all score‐based tests for parameter instability which are applied to the covariates in all data sets should result in significant p‐values. For each simulation scenario and model, 1000 simulation samples (i.e. repetitions) were generated. We denote the percentage of simulated samples for which the p‐value of the score‐based test is smaller than .05 as the power of the score‐based test.

For each of the simulated samples, ordinal factor models are fitted with the WLS estimator using the lavaan package (Rosseel, 2012) and (multidimensional) GRMs are fitted via FI estimation, namely the MML estimator, using the mirt package (Chalmers, 2012). With FI estimation, the unidimensional model is fitted via the EM algorithm and the multidimensional model is fitted via the MH‐RM algorithm. Each of the fitted models is tested for parameter instability using the *maxLM*, *DM* and *CvM* test statistics on $Z_{\mathrm{num}}$, the *WDMo* and *maxLMo*test statistics on $Z_{\mathrm{ord}}$ and the *LMuo* test statistic on $Z_{\mathrm{cat}}$.

We further conduct additional simulations with data that do not exhibit DIF, that is, the values of the covariates were simulated randomly. This means that all score‐based tests for parameter instability which are applied to the covariates in all data sets should not result in significant p‐values. We denote the percentage of simulated samples for which the p‐value of the score‐based test is smaller than .05 as the Type I error rate of the score‐based test.

To see how the approximated scores of the ordinal factor model are distributed, we additionally simulate two data sets to fit the unidimensional model (Figure A1). One data set has binary response variables and the other data set has response variables with four ordered response categories. We simulate two other data sets to fit the multidimensional model (Figure A2). We then use three different approaches to fit the models to the data: ordinal factor analysis (LI estimation), FI estimation and GEE estimation (see Appendix S1). Subsequently, the models scores are estimated for each model for each data set. The correlations of the model score contributions of each parameter in the respective model are shown in Table A1 (for the unidimensional model) and A2 (for the multidimensional model).

### 3.1 Results

The means of the NCDIF values in Table A3 show that DIF effect sizes on one item are considerably lower if only the discrimination parameters differ between the focal and the reference group. This is reflected in the power results of the simulation for both the unidimensional and the multidimensional model. However, if only the thresholds of an item differ between focal and reference group, the DIF effect size of that item is similar to the case in which all parameters differ. From this result, we deduce that the power of the score‐based test in the first simulation scenario (all parameters differ) most likely does not differ significantly from a scenario in which only the threshold values of all items differ. The second simulated scenario for parameter fluctuation thus consists of only the threshold parameters of one item (i.e. 20% DIF in the unidimensional model), respectively, of two items (i.e. 22% DIF in the multidimensional model) differing between the focus and reference groups.

**FIGURE A4:**
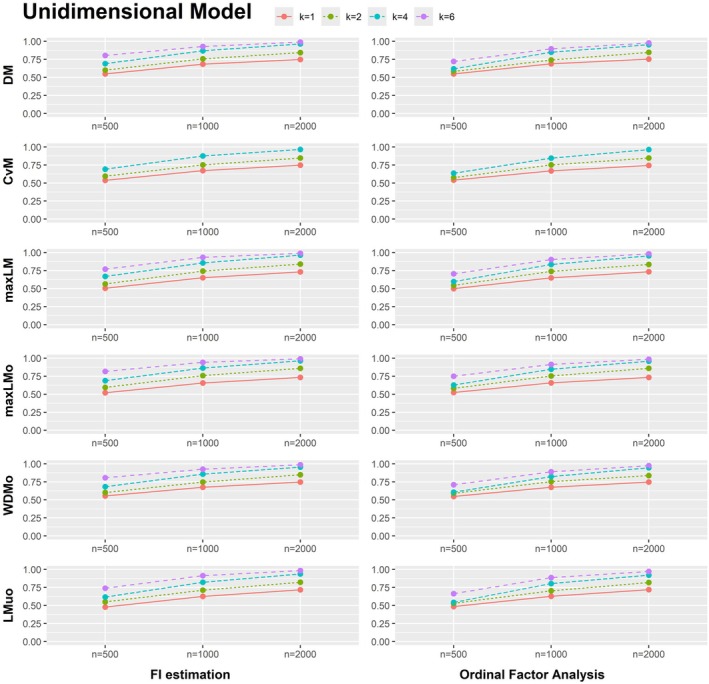
Power of score‐based test for the unidimensional graded response model (GRM) given fluctuation with respect to the threshold parameters of the first item $\tau_{1k}$.

**FIGURE A5:**
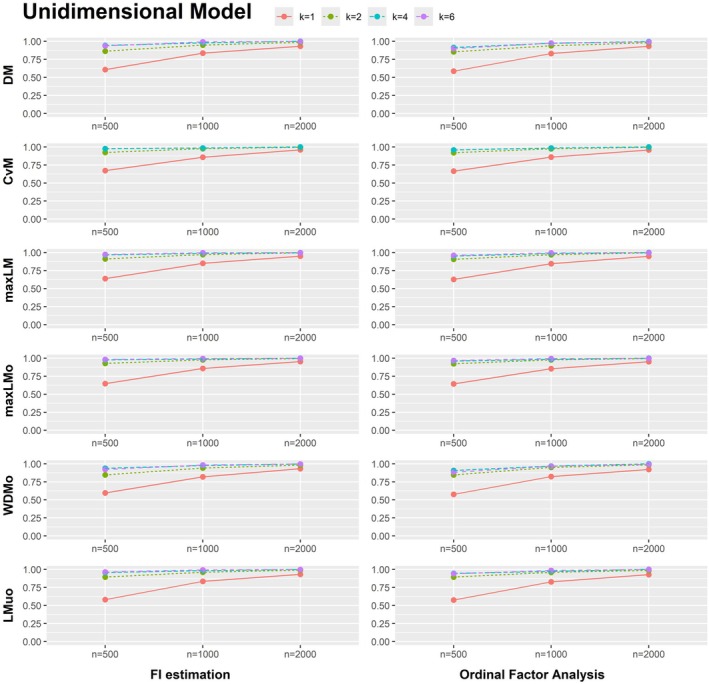
Power of score‐based test for the unidimensional graded response model (GRM) given fluctuation with respect to the discrimination parameters $\lambda_{i}$.

The results of the simulations with data based on the unidimensional model (see Figure A1) show that power generally increases with sample sizes and the number of response categories. For the proposed score‐based tests for ordinal factor models as well as for tests based on GRMs fitted via FI estimation, power lies between 98% and 100% when there is parameter fluctuation with respect to all model parameters. Figure A4shows that given fluctuation with respect to the threshold parameters of the first item $\tau_{1k}$ only, sample sizes of at least n=2000 are needed for k=4 thresholds and sample sizes of at least n=1000 are needed for k=6 thresholds to achieve power of over 90% for all test statistics. For the simulated data sets with parameter fluctuation with respect to the discrimination parameters $\lambda_{j}$, power results for both ordinal factor models and for GRMs fitted via FI estimation are shown in Figure A5. For k=1, sample sizes of n=2000 are needed to achieve power of over 90% for all tests statistics. In general, with respect to power, the score‐based test does not perform better for models fitted via FI estimation as compared to ordinal factor models.

Type I error results for the unidimensional model are generally within the expected range of 3% and 6% for all test statistics for ordinal factor models and for GRMs fitted via FI estimation for all sample sizes and numbers of thresholds. This indicates that the score‐based test for ordinal factor analysis performs equally well as for the GRMs fitted via FI estimation when estimating unidimensional IRT models. The computation times for fitting the unidimensional GRM via FI estimation (using the EM algorithm) and the ordinal factor models are very similar (see Table A4).

**FIGURE A6:**
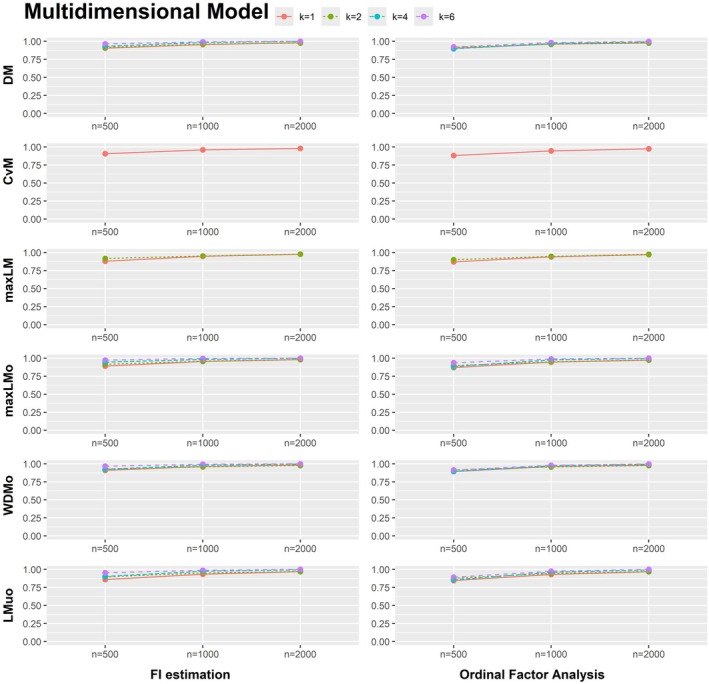
Power of score‐based test for the multidimensional graded response model (GRM) given fluctuation with respect to the threshold parameters of the first two items, that is, $\tau_{1k}$ and $\tau_{2k}$.

**FIGURE A7:**
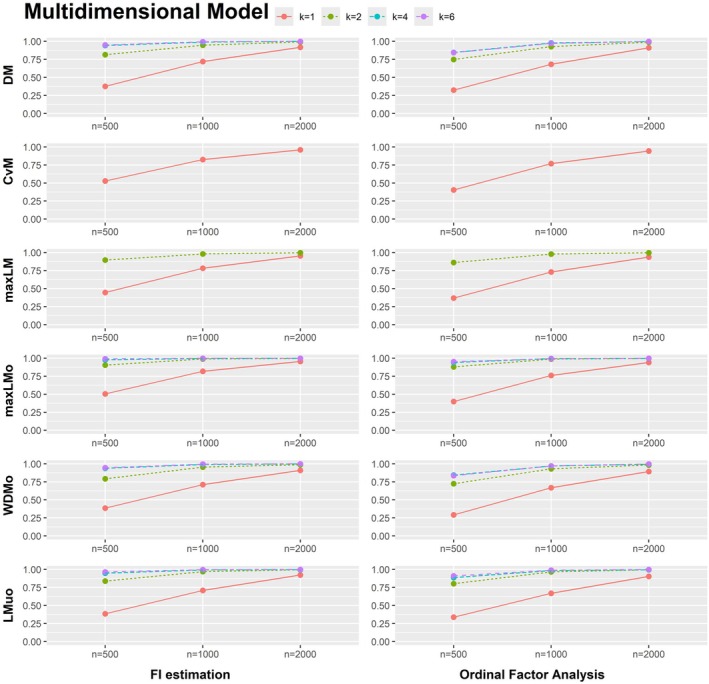
Power of score‐based test for the multidimensional graded response model (GRM) given fluctuation with respect to the discrimination parameters $\lambda_{i}$.

**FIGURE A8:**
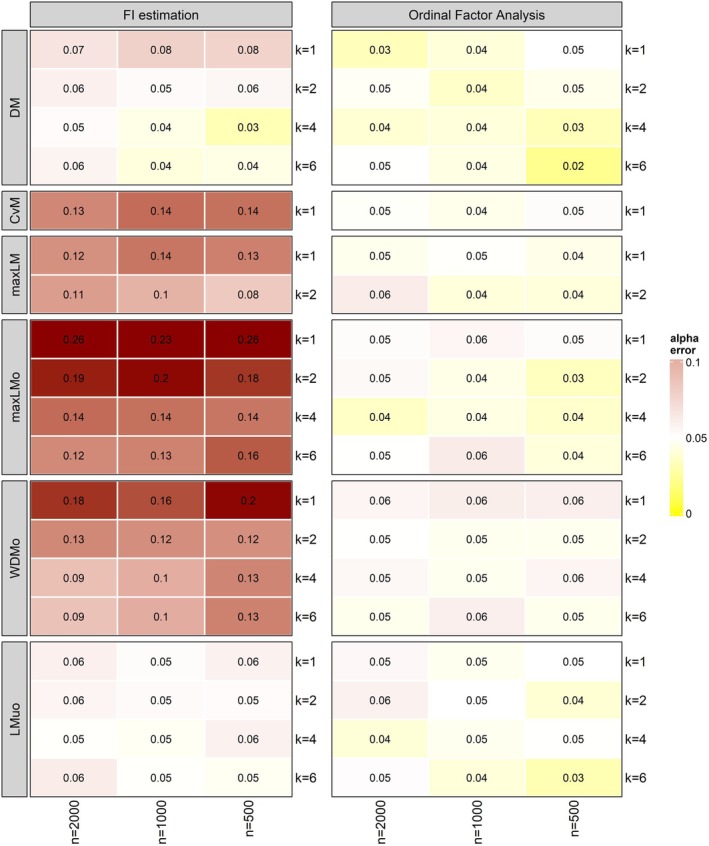
Type I errors of score‐based test for the multidimensional graded response model (GRM). Note that for the *CvM* test statistic, there are no critical values implemented in the strucchange package for models with more than 25 parameters. This also applies for the *maxLM* test statistic for models with more than 40 parameters. Therefore, models with more than 1 (for *CvM*) and 2 (for *maxLM*) threshold parameters are not shown.

Computation times for fitting the multidimensional model (see Figure A2) are much higher for FI estimation (using the MH‐RM algorithm) compared to ordinal factor analysis with LI estimation (see Table A5), highlighting the benefits of ordinal factor analysis in this setting. The results also show that high power (100%) is achieved for both ordinal factor models and for GRMs fitted via FI estimation when there is parameter fluctuation with respect to all model parameters. Power results for the data sets with parameter fluctuation with respect to only the threshold parameters of item 1 and 2 are shown in Figure A6. Interestingly, the multidimensional model outperforms the unidimensional model in this simulation scenario. Here, sample sizes of n=1000 suffice for models with two response categories to achieve power of over 90% for all test statistics. The power results of the score‐based test when the discrimination parameters $\lambda_{j}$ of all items differ between the focal and the reference group are shown in Figure A7. When only the discrimination parameters differ in data sets of n=500 and k=1, power lies between 29.2% and 52.8%. For data sets with k=2, power is at least 72.5%. Power results are generally very similar between ordinal factor models and for GRMs fitted via FI estimation. However, there are considerable differences between these two types of models regarding the Type I error (see Figure A8). Type I error rate is higher for the score‐based tests applied to GRMs fitted via FI estimation. This is particularly the case for the *CvM*, *maxLM*, *maxLMo* and *WDMo* test statistics.

The correlations of the model scores for the unidimensional model in Table A1 show that the score contributions of the model fitted with the GEE approach correlate negatively with the scores of the models fitted with the LI (i.e. ordinal factor analysis) or the FI approach. This is because of the definition of the Δ‐matrix for GEE estimation, where $\frac{\partial \text{vec}(\nu )(\theta )}{\partial \theta }$ contains negative values (see Equation 19 in Appendix S1). The approximated model score contributions of the ordinal factor model correlate strongly with the score contributions of the model fitted with the GEE approach. The parameter estimates are expected to differ between the two approaches; therefore, perfect correlations of the score contributions are not expected. Interestingly, the correlations of the model score contributions from the GEE approach with the score contributions from the FI approach are lower for discrimination parameters and higher for threshold parameters. The correlations of the model score contributions from the LI approach with those from the FI approach are generally a bit lower than those from the LI approach with those from the GEE approach. The correlations of the model scores for the multidimensional model (Table A2) show a very similar pattern.

## 4 REAL DATA APPLICATION

We demonstrate the application of score‐based tests with (multidimensional) GRMs using data obtained from the LISS (Longitudinal Internet studies for the Social Sciences) panel administered by Centerdata (Tilburg University, the Netherlands). LISS is a longitudinal survey conducted annually, covering topics such as employment, education, income, housing and personality traits (Scherpenzeel & Das, 2010). We analyse the data from four survey waves that were conducted in 2008, 2009, 2011 and 2013. In the survey waves of 2010 and 2012, certain application‐relevant items were not included. A total of 2893 individuals participated across all four waves of the survey. Our analysis focuses on five items from the Satisfaction with Life (SL) scale (Diener et al., 1985), which assesses life satisfaction. We excluded any cases that did not provide responses to all five items, resulting in a final sample size of 2888 individuals. The items were rated on a seven‐point response scale. The specific wording of these items is displayed in Table A6.

**FIGURE A9:**
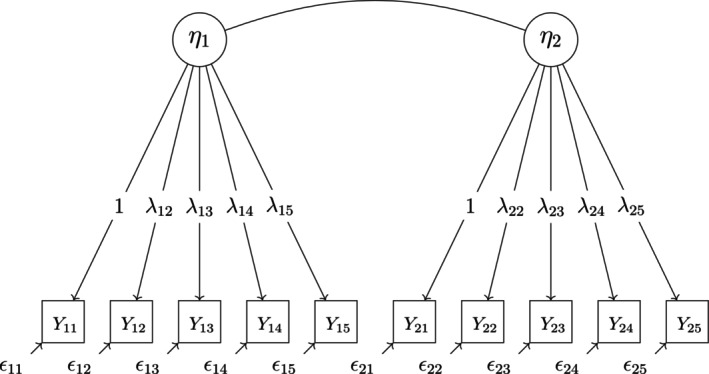
Real data application model 2: multidimensional graded response model (GRM) with two latent state variables and five items on two time points.

**FIGURE A10:**
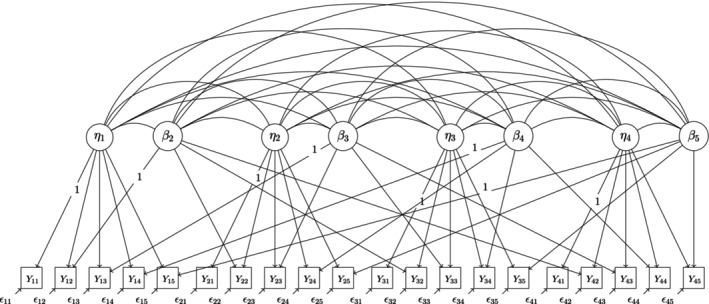
Real data application model 3: probit multistate IRT model with latent item effect variables for graded responses (PIEG) with four latent state variables ($\eta_{t}$), four latent item effect variables ($\beta_{i}$) and five items on three time points.

We apply three different models of different sizes to the data. Model 1 has the same unidimensional GRM model structure used in the simulation (see Figure A1). The five items $Y_{j}$ represent the SL scale in the first survey wave. Model 2 has a multidimensional GRM model structure with correlated latent variables and is shown in Figure A9. The items $Y_{tj}$ represent the SL scale in survey waves one (t=1) and two (t=2). Model 3 is a *probit multistate IRT model with latent item effect variables for graded responses* (PIEG) in which one reference latent state variable $\eta_{t}$ is assumed for every time point of measurement and one latent item effect variable $\beta_{i}$ is defined for every item but the reference item (here: j=1). In this model, the variances and covariances of the latent state variables, as well as the latent item effect variables and the covariances between them, are estimated. The discrimination parameters in the model are all fixed at 1 (Classe & Steyer, 2023). The model is shown in Figure A10.

We fit each model using three different estimation methods: ordinal factor analysis (using the WLS estimator), FI estimation (Model 1 via the EM algorithm, and Model 2 and Model 3 via the MH‐RM algorithm) and common factor analysis. For common factor analysis, we use the robust maximum likelihood (MLR) estimator, since here the model fit statistics are corrected for the non‐normality of the response variables (Li, 2016).

For every fitted model, we apply the score‐based test with respect to three different background variables representing general characteristics of households and household members that participate in the LISS panel: Gender (categorical: “Female”, “Male” and “Other”), urban character of place of residence (ordinal: five categories from “extremely urban” to “not urban”) and individual age (metric). We do not assume an impact of any of the covariates on satisfaction with life. This is mainly due to methodological considerations. We do not want to specify an anchor item as we assume that the item characteristics of all five items may differ across the subgroups defined by the covariates. For the categorical covariate, we use the *LMuo*; for the ordinal covariate, we use the *WDMo*; and for the metric covariate, we use the *DM* test statistic. All three test statistics can be used with large models as they obtain their critical values through closed‐form solutions of certain functions instead of default tables.

We analyse the fitted models with respect to the degree of model fit and the computation time of the model fitting process and apply the score‐based test using the outlined covariates. Furthermore, for each model, we analyse the time needed to compute the empirical fluctuation process, which includes the computation of the model scores.

### 4.1 Results

The results of the real data application displayed in Table A7 show that computation time increases when fitting larger ordinal factor models compared to smaller ones. However, compared to the considerable increase in computation time for fitting the GRMs via FI estimation, the increase in computation time for larger ordinal factor models is marginal. This agrees with the simulation results shown in Tables A4 and A5 and shows that FI estimation is not computationally efficient for models with two or more non‐orthogonal latent variables. Compared to FI estimation, ordinal factor analysis is computationally efficient, even for large models. When it comes to the results of the score‐based tests, models fitted via FI estimation are very similar to ordinal factor models, at least for model 1 and model 2. For model 3, all p‐values for the score‐based test are smaller than 2.20E‐16. Also, computing the empirical fluctuation process is particularly expensive for model 3 when fitted via FI estimation.

Comparing the results of the common factor models with the ordinal factor models shows that common factor analysis is computationally faster than ordinal factor analysis, especially for large models. These results are also shown in Table A7. Also, the model fit estimation results of common factor analysis (using the MLR estimator) are similar to those of ordinal factor analysis. However, there are considerable discrepancies with respect to the results of the score‐based tests, particularity for the categorical and metric covariates for all model sizes. Note that in using common factor models for categorical data, model misspecification is introduced as, for instance, no threshold parameters are estimated.

## 5 DISCUSSION

The results of our simulations show that score‐based tests for parameter instability perform equally well for unidimensional GRMs fitted via FI estimation and for ordinal factor models. As there are no considerable differences regarding computation time, we conclude that fitting univariate IRT models and testing them for parameter instability is equally convenient using FI estimation or ordinal factor analysis.

However, the results of the simulation regarding the multidimensional model show that there are considerable differences in computation times when fitting the model via FI estimation (using the MH‐RM algorithm) compared to ordinal factor analysis. The limited information method of ordinal factor analysis is 32–91 times faster than the MH‐RM algorithm. The power results indicate that the proposed score‐based test for unidimensional GRMs as well as for multidimensional GRMs implemented via ordinal factor models performs equally well as tests based on unidimensional GRMs fitted via FI estimation. However, when it comes to multidimensional GRMs, there are considerable specificity problems of the score‐based test when applied to models fitted via FI estimation in contrast to ordinal factor analysis. Debelak et al. (2024) point out that increased Type I errors of the score‐based test when applied to models fitted via FI estimation could be due to numerical inaccuracies of the MH‐RM algorithm. Additional fine‐tuning of the implementation of the algorithm in the mirt package may help to obtain accurate Type I error rates.

The distribution of the approximated scores of the ordinal factor model are generally very similar to the scores from the GEE estimation method. Note that, unlike LI estimation, the GEE estimation method optimizes the model scores to estimate the model's parameters. This takes a very long time, especially for multidimensional non‐binary models. The fact that the scores are distributed similarly to the model scores estimated with the method proposed in this paper indicates that our approach is, in fact, a valid approximation and thus a computationally efficient alternative when it comes to parameter instability tests.

Note that the score‐based test may also be applicable for GRMs fitted via PML (which is also a LI method). However, in their application Wang, Strobl, et al. (2018) focused on unidimensional two‐parameter normal ogive models for dichotomous response variables.

The real data applications show that the results for the score‐based tests are very similar for unidimensional ordinal factor models and models fitted via FI estimation. This matches the results of the simulation. For very large models, however, the discrepancy between score‐based tests applied to ordinal factor models and GRMs fitted via FI estimation is considerable. Additionally, it appears that score‐based tests for parameter instability produce different results for ordinal factor analysis compared to common factor analysis. We therefore conclude that ordinal factor analysis should be preferred over common factor analysis and GRMs fitted via FI estimation when testing for parameter instability in multidimensional GRMs.

Note that, within our simulated samples, the covariates Z are always independent from the latent variable distribution ξ (in both the unidimensional and the multidimensional case). This implies that only single‐group MIRT models without differences in the latent variable between subgroups are considered. Also for the real data applications in this paper, we assume independence of the covariates from the latent variable distribution. Future research might investigate the performance of the score‐based test for multiple‐group ordinal factor models.

### 5.1 Model‐based recursive partitioning

Methods based on the score‐based test can be very helpful in scenarios where there are a multitude of metric, ordinal or categorical covariates potentially causing DIF. In such contexts, data‐driven methods such as Model‐Based Recursive Partitioning (MOB; Zeileis et al., 2008) prove valuable for identifying subgroups in which DIF is present. This algorithm repeatedly splits a sample into subgroups based on covariates $Z_{r}$ in $Z_{1},\text{…},Z_{R}$ (referred to as partitioning variables) to form a decision tree (see Breiman et al., 1984). The score‐based test for parameter instability can be used in such a recursive partitioning algorithm to account for parameter instability. When parameter instability is detected in a tree node during the partitioning process, that is, the score‐based test for one of the partitioning variables falls below a predefined significance level, the partitioning variable $Z_{r^{*}}$ associated with the smallest p‐value is selected for partitioning. The unique value of a partitioning variable that maximizes the respective score‐based test statistic can be used as a split point (see Arnold et al., 2021). The MOB algorithm continues to partition different subgroups until the stopping criteria are met. This is usually the case when there is no more significant instability in a node or when the subsample in a node becomes too small to fit the model. However, the application of MOB in conjunction with ordinal factor models is not yet implemented in the available R packages. The quick computation of MOB trees for MIRT models may, among other things, be relevant for the estimation of unbiased latent variable scores (Classe & Kern, 2024). Thus, future research should further investigate the application of MOB to ordinal factor models, building on the technique proposed in this paper.

### 5.2 Outlook

The efficient computation of individual model scores for MIRT models is not only useful for efficient computation of parameter instability tests. The proposed method may also be used to compute robust test statistics based on sandwich covariance matrices (Zeileis, 2006). Such robust corrections are already widely used in structural equation modelling with complete (Savalei, 2014) or incomplete (Savalei & Rosseel, 2022) data. Another possible area of application is model selection of non‐nested models via Vuong tests, since the Vuong test statistics are generally calculated on the basis of the individual model scores (Schneider et al., 2020). With the method proposed in this paper, such advances can be extended to ordinal factor models.

## AUTHOR CONTRIBUTIONS

**Franz Classe:** conceptualization; investigation; writing – original draft; methodology; visualization; software; validation; project administration; formal analysis. **Rudolf Debelak:** writing – review and editing; methodology; project administration. **Christoph Kern:** writing – review and editing; methodology; project administration.

## CONFLICT OF INTEREST STATEMENT

The authors declare that they have no known competing financial interests or personal relationships that could have appeared to influence the work reported in this paper.

## Supporting information


Appendix S1

## Data Availability

The data that support the findings of this study are openly available in OSF at https://osf.io/hmwpc/?view_only=69ed2919e7a64db2b0354f99243c307c.

## Footnotes

1 In Debelak and Strobl (2019b), other examples of ψ can be found.

## APPENDIX A TABLES

**TABLE A1:** Correlation of model scores for a unidimensional GRM (see Figure A1) with binary and non‐binary (four ordered categories) response variables fitted on a simulated data set with n=2000 respondents. The model scores of three different fitted models are compared: ${\mathrm{SC}}_{\mathrm{OFA}}$ meaning the approximated scores for a ordinal factor model, ${\mathrm{SC}}_{\mathrm{FI}}$ meaning the scores for a model fitted with FI estimation and ${\mathrm{SC}}_{\mathrm{GEE}}$ meaning the scores of a model fitted with GEE (see Appendix S1).

	$\mathrm{Cor}({\mathrm{SC}}_{\mathrm{OFA}},{\mathrm{SC}}_{\mathrm{GEE}})$		$\mathrm{Cor}({\mathrm{SC}}_{\mathrm{FI}},{\mathrm{SC}}_{\mathrm{GEE}})$		$\mathrm{Cor}({\mathrm{SC}}_{\mathrm{OFA}},{\mathrm{SC}}_{\mathrm{FI}})$	
	Binary	Non‐binary	Binary	Non‐binary	Binary	Non‐binary
$\mathrm{Var}(\eta_{1})$	.94	.97	.99	.95	.93	.96
$\lambda_{2}$	.91	.94	.88	.85	.96	.94
$\lambda_{3}$	.94	.93	.93	.80	.98	.91
$\lambda_{4}$	.94	.90	.96	.73	.99	.87
$\lambda_{5}$	.92	.91	.92	.68	.97	.82
$\tau_{11}$	−.98	−.92	−.96	−.94	.99	.76
$\tau_{12}$		−.95		−.97		.90
$\tau_{13}$		−.92		−.99		.90
$\tau_{21}$	−.99	−.94	−.98	−.96	1.00	.84
$\tau_{22}$		−.97		−.99		.95
$\tau_{23}$		−.93		−1.00		.91
$\tau_{31}$	−.99	−.93	−.99	−.96	1.00	.81
$\tau_{32}$		−.97		−.99		.96
$\tau_{33}$		−.92		−.98		.85
$\tau_{41}$	−.97	−.90	−.96	−.99	1.00	.84
$\tau_{42}$		−.97		−.98		.92
$\tau_{43}$		−.92		−.94		.76
$\tau_{51}$	−1.00	−.91	−.99	−.96	1.00	.78
$\tau_{52}$		−.97		−.98		.95
$\tau_{53}$		−.90		−.92		.69

*Note*: White color means perfect (positive or negative) correlation. Shades of red indicate a deviation from the perfect correlation.

**TABLE A2:** Correlation of model scores for a multidimensional GRM (see Figure A2) with binary and non‐binary (four ordered categories) response variables fitted on a simulated data set with n=2000 respondents. The model scores of three different fitted models are compared: ${\mathrm{SC}}_{\mathrm{OFA}}$ meaning the approximated scores for a ordinal factor model, ${\mathrm{SC}}_{\mathrm{FI}}$ meaning the scores for a model fitted with FI estimation, and ${\mathrm{SC}}_{\mathrm{GEE}}$ meaning the scores of a model fitted with GEE (see Appendix S1).

	$\mathrm{Cor}({\mathrm{SC}}_{\mathrm{OFA}},{\mathrm{SC}}_{\mathrm{GEE}})$		$\mathrm{Cor}({\mathrm{SC}}_{\mathrm{FI}},{\mathrm{SC}}_{\mathrm{GEE}})$		$\mathrm{Cor}({\mathrm{SC}}_{\mathrm{OFA}},{\mathrm{SC}}_{\mathrm{FI}})$	
	Binary	Non‐binary	Binary	Non‐binary	Binary	Non‐binary
$\mathrm{Var}(\eta_{1})$	.97	.96	.92	.71	.96	.83
$\mathrm{Var}(\eta_{2})$	.91	.95	.80	.67	.83	.80
$\mathrm{Var}(\eta_{3})$	.93	.97	.80	.89	.88	.89
$\mathrm{Cov}(\eta_{1},\eta_{2})$	.89	.85	.91	.74	.93	.88
$\mathrm{Cov}(\eta_{1},\eta_{3})$	.91	.84	.90	.85	.95	.93
$\mathrm{Cov}(\eta_{2},\eta_{3})$	.88	.90	.85	.84	.91	.93
$\lambda_{12}$	.93	.91	.85	.51	.87	.68
$\lambda_{13}$	.92	.87	.89	.77	.87	.89
$\lambda_{22}$	.85	.88	.87	.72	.93	.86
$\lambda_{23}$	.92	.87	.65	.54	.68	.71
$\lambda_{32}$	.93	.88	.90	.74	.93	.81
$\lambda_{33}$	.91	.85	.76	.73	.90	.79
$\tau_{11}$	−.98	−.88	−.98	−.97	1.00	.79
$\tau_{12}$		−.92		−.98		.88
$\tau_{13}$		−.89		−.94		.76
$\tau_{21}$	−.98	−.89	−.98	−.94	1.00	.74
$\tau_{22}$		−.93		−.98		.90
$\tau_{23}$		−.90		−.91		.71
$\tau_{31}$	−.99	−.84	−.99	−.88	1.00	.64
$\tau_{32}$		−.83		−.90		.71
$\tau_{33}$		−.83		−.94		.82
$\tau_{41}$	−.95	−.88	−.94	−.94	.99	.71
$\tau_{42}$		−.94		−.99		.93
$\tau_{43}$		−.88		−.98		.80
$\tau_{51}$	−.88	−.88	−.88	−.98	.97	.81
$\tau_{52}$		−.95		−.99		.94
$\tau_{53}$		−.89		−.95		.74
$\tau_{61}$	−.97	−.87	−.97	−.94	.99	.71
$\tau_{62}$		−.93		−.99		.92
$\tau_{63}$		−.88		−.92		.65
$\tau_{71}$	−.81	−.94	−.91	−1.00	.84	.92
$\tau_{72}$		−.93		−1.00		.93
$\tau_{73}$		−.88		−1.00		.87
$\tau_{81}$	−.98	−.90	−.98	−1.00	1.00	.90
$\tau_{82}$		−.85		−1.00		.85
$\tau_{83}$		−.82		−.99		.81
$\tau_{91}$	−.94	−.90	−.92	−1.00	.99	.90
$\tau_{92}$		−.88		−.99		.88
$\tau_{93}$		−.79		−.99		.76

*Note*: White color means perfect (positive or negative) correlation. Shades of red indicate a deviation from the perfect correlation.

**TABLE A3:** Means of noncompensatory DIF (NCDIF) effect sizes for Item 2. Results for 1000 simulated samples with sample size of n=1000. Modes: “all” for all parameters differ, “thresholds” for only thresholds differ and “betas” for only discrimination parameters differ between focal group and reference group.

Mode		Model	
		Unidimensional	Multidimensional
All	k=1	.05	.14
All	k=2	.18	.48
All	k=4	.54	1.75
All	k=6	1.20	3.65
Thresholds	k=1	.04	.13
Thresholds	k=2	.15	.49
Thresholds	k=4	.47	1.65
Thresholds	k=6	1.02	3.46
Lambdas	k=1	.01	.01
Lambdas	k=2	.05	.04
Lambdas	k=4	.15	.14
Lambdas	k=6	.31	.31

**TABLE A4:** Computation time in seconds for fitting the unidimensional GRM given no parameter fluctuation in the data. FI, meaning full information estimation, corresponds to model estimation with the MML estimator. LI, meaning limited information estimation, corresponds to ordinal factor analysis with the WLS estimator.

	n=500		n=1000		n=2000	
	FI	LI	FI	LI	FI	LI
k=1	.19	.19	.20	.21	.21	.23
k=2	.23	.20	.25	.22	.27	.26
k=4	.31	.25	.36	.27	.38	.31
k=6	.41	.30	.47	.31	.51	.38

**TABLE A5:** Computation time in seconds for fitting the multidimensional GRM given no parameter fluctuation in the data. FI, meaning full information estimation, corresponds to model estimation with the MML estimator. LI, meaning limited information estimation, corresponds to ordinal factor analysis with the WLS estimator.

	n=500		n=1000		n=2000	
	FI	LI	FI	LI	FI	LI
k=1	12.80	.39	17.42	.46	21.02	.39
k=2	14.04	.42	19.53	.40	27.40	.42
k=4	18.38	.52	26.65	.47	40.92	.50
k=6	22.48	.64	50.66	.84	57.21	.63

**TABLE A6:** Life satisfaction scale items as asked in the LISS panel.

Text: Below are five statements with which you may agree or disagree. Using the 1–7 scale below, indicate your agreement with each item by placing the appropriate number on the line preceding that item. Please be open and honest in your responding.	
Item	Wording
i=1	In most ways, my life is close to my ideal
1	The conditions of my life are excellent
2	I am satisfied with my life
3	So far I have gotten the important things I want in life
4	If I could live my life over, I would change almost nothing

**TABLE A7:** Results of the real data application.

			Model 1 (unidim.)	Model 2 (multidim.)	Model 3 (PIEG)
Ordinal factor analysis	Number of parameters		35	63	156
	RMSEA		.127	.127	.051
	Score‐based test p‐value	Categorical	1.04E‐05	2.22E‐04	.017
		Ordinal	.918	.694	.689
		Metric	.165	.008	.014
	Computation time	Model	.345	.913	6.189
		Scores	.063	.109	.325
GRM: FI estimation	Number of parameters		35	63	156
	RMSEA		0	0	0
	Score‐based test p‐value	Categorical	3.92E‐06	4.02E‐05	0
		Ordinal	.181	.445	0
		Metric	.197	.002	0
	Computation time	Model	.541	88.428	301.546
		Scores	.287	15.506	1074.466
Common factor analysis	Number of parameters		10	13	56
	RMSEA		.099	.122	.043
	Score‐based test p‐value	Categorical	.002	.266	.424
		Ordinal	.227	.453	.770
		Metric	.014	.000	.040
	Computation time	Model	.186	.142	.514
		Scores	.089	.194	.247
