# Supplementary material for: Score‐based tests for parameter instability in ordinal factor models
Source: Br J Math Stat Psychol. 2025 Apr 23;78(3):996–1024. doi: 10.1111/bmsp.12392 (PMC12516115; doi:10.1111/bmsp.12392)
Supplement: Supplementary file 1 — Appendix S1 [file BMSP-78-996-s001.pdf]

Score-Based Tests for Parameter Instability in Ordinal Factor Models: Technical Appendix

Score-Based Tests for Parameter Instability in Ordinal Factor Models: Technical Appendix

Generalized Estimating Equations (GEE) for Ordinal Factor Analysis

In the introduction of the main text, we highlight that parameter estimation for multidimensional IRT (MIRT) models via polychorics is also referred to as limited-information (LI) estimation, as it only uses information from bivariate relations of the observed variables. We then present the WLS estimator, as originally introduced by Muthén (1983, 1984). An alternative LI estimation method is presented in this chapter. To our knowledge, this estimation method had not yet been described or implemented for non-binary data by prior research and is thus discussed in this section. Let $\boldsymbol{Y}$ be a $p \times 1$ vector of ordered observed variables. For simplicity, we assume that all ordered observed variables have l response categories denoted by the index k. In an ordinal factor model (Maydeu-Olivares, 2005), a continuous, normally distributed latent observed variable Y_j^* is assumed to underlie each observed ordered variable $Y_j \, \forall \, j = 1, \ldots, p,$. Just as in a conventional factor model, a linear measurement structure is assumed. That is

$$\boldsymbol{Y}^* = \boldsymbol{\lambda}'\boldsymbol{\xi} + \boldsymbol{\epsilon}, \tag{1}$$

where $\boldsymbol{\xi}$ is the the $m \times 1$ vector of continuous latent variables, and $\boldsymbol{\lambda}$ is the $m \times p$ matrix of discrimination parameters (also referred to as factor loadings). Also, $\boldsymbol{\epsilon}$ is a $p \times 1$ vector of residuals. In the following, we refer to $Cov(\boldsymbol{Y}^*)$ as the *model implied* covariance matrix.

The latent observed variable Y_j is related to the observed ordered variable via a threshold relation, that is

$$Y_j = k_j \text{ if } \tau_{j(k-1)} < y_j^* \leq \tau_{jk}. \tag{2}$$

This means that a respondent chooses a response category k_j when the respondent's latent response value y_j^* on item j lies between the thresholds $\tau_{j(k-1)}$ and τ_{jk}, where

$\tau_{j0} = -\infty$ and $\tau_{jl} = +\infty$.

The model parameter vector θ contains all freely estimated model parameters. That includes the threshold parameters τ_{jk} for all items $j = 1, \dots p$, belonging to the item categories $k = 1, \dots, l-1$. Note that, for each item, there is one threshold parameter less than there are item categories. Furthermore, θ contains all freely estimated discrimination parameters λ_{qj} for all latent variables $q = 1, \dots, m$, and all items $j = 1, \dots, p$, as well as all freely estimated latent variable variances and covariances, such that

$$\begin{aligned}\theta = \{&\tau_{11}, \dots, \tau_{pl}, \lambda_{11}, \dots, \lambda_{mp}, \\ &Var(\xi_1), \dots, Var(\xi_m), \\ &Cov(\xi_1, \xi_2), \dots, Cov(\xi_{m-1}, \xi_m)\}.\end{aligned} \tag{3}$$

In the original approach, a three-stage generalized least squares procedure for parameter estimation was proposed. However, Reboussin and Liang (1998) claim that this estimation method may not perform well for small sample sizes and large numbers of indicator variables. They therefore propose an alternative estimation procedure based on an quadratic estimating equations. Muthén (1997) referred to this method as the *Generalized Estimating Equation* (GEE) approach.

For the purpose of finding a computationally feasible way to compute model scores for ordinal factor models, the GEE approach seems very promising. Parameter estimation via GEEs is closely related to the idea of the *M-estimator* (Stefanski & Boos, 2002), which is an estimator $\hat{\theta}$ that satisfies

$$\sum_{i=1}^{n} \psi(\boldsymbol{y}_i, \hat{\theta}) = \mathbf{0}, \tag{4}$$

where $\boldsymbol{y}_i$ is the $p \times 1$ vector of observed responses of individual i.

From Muthén (1997) and Reboussin and Liang (1998), we derive that the (model

implied) true mean μ_j of the observed variable Y_j is

$$\mu_j(\theta) = \mu_j = E(Y_j) = \sum_{k=1}^{l} k \cdot P(Y_j = k). \tag{5}$$

The true category probabilities of Y_j can be computed on the basis of the threshold parameters τ_{jk}, such that

$$\begin{aligned} P(Y_j = k) &= \Phi(\tau_{j1}) \text{ if } k = 1, \\ P(Y_j = k) &= \Phi(\tau_{jk}) - \Phi(\tau_{j;k-1}) \text{ if } 1 < k < l, \\ P(Y_j = k) &= 1 - \Phi(\tau_{j(l-1)}) \text{ if } k = l. \end{aligned} \tag{6}$$

The true joint probabilitiy $P(Y_j = k, Y_s = h)$ and thus the true joint expectation $E(Y_j Y_s)$, can be calculated as follows

$$\begin{aligned} P(Y_j = k, Y_s = h) =& \Phi_2(\tau_{jk}, \tau_{sh}, \sigma^*_{js}) - \\ & \Phi_2(\tau_{j;k-1}, \tau_{sh}, \sigma^*_{js}) - \\ & \Phi_2(\tau_{jk}, \tau_{s;h-1}, \sigma^*_{js}) - \\ & \Phi_2(\tau_{j;k-1}, \tau_{s;h-1}, \sigma^*_{js}), \\ E(Y_j Y_s) =& \sum_{k=1}^{l} \sum_{h=1}^{g} k \cdot h \cdot P(Y_j = k, Y_s = h), \end{aligned} \tag{7}$$

where σ^*_{js} are elements in the model implied covariance matrix, i.e. $\sigma^*_{js} = Cov(Y^*_{js})$. This definition is derived from Equation 4 in Olsson (1979).

Furthermore, let the second order moment of Y_j and Y_s be

$$\sigma_{js} = E(Y_j Y_s) - \mu_j \mu_s. \tag{8}$$

For the case of non-binary observed variables, the first order moments of Y_j are made up from the indicator variables $1_{Y_j > k} \; \forall \; k = 1, \ldots, l-1$. The true mean of $1_{Y_j > k}$ is

$$\nu_{jk} = E(1_{Y_j > k}) = P(Y_j > k) = P(Y^*_j > \tau_{jk}) = \Phi(-\tau_{jk}). \tag{9}$$

Note that $1_{Y_j>k} = Y_j$ in the special case of dichotomous observed variables (as reported in Muthén, 1997; Reboussin & Liang, 1998).

To fit an ordinal factor models via GEEs, first and second order individual empirical moments are defined. Let the $(l-1) \times p$ matrix $\mathbf{1}_{\mathbf{y}_i}$ contain the first order empirical moments of individual i, that is

$$\mathbf{1}_{\mathbf{y}_i} = \begin{pmatrix} 1_{y_{i1}>1} & \cdots & 1_{y_{ip}>1} \\ 1_{y_{i1}>2} & \cdots & 1_{y_{ip}>2} \\ \vdots & \ddots & \vdots \\ 1_{y_{i1}>l-1} & \cdots & 1_{y_{ip}>l-1} \end{pmatrix}. \tag{10}$$

The first order moments of $\boldsymbol{Y}$ are in the $(l-1) \times p$ matrix

$$\boldsymbol{\nu} = \begin{pmatrix} \nu_{11} & \nu_{21} & \cdots & \nu_{p1} \\ \nu_{12} & \nu_{22} & \cdots & \nu_{p2} \\ \vdots & \ddots & \ddots & \vdots \\ \nu_{1;l-1} & \nu_{2;l-1} & \cdots & \nu_{p;l-1} \end{pmatrix}. \tag{11}$$

Moreover, let $\boldsymbol{\sigma}$ be a vector of second order moments of $\boldsymbol{Y}$. This vector includes all non-redundant, off-diagonal elements of the true covariance matrix of $\boldsymbol{Y}$, i.e.

$$\boldsymbol{\sigma} = \begin{pmatrix} \sigma_{12} \\ \sigma_{13} \\ \vdots \\ \sigma_{p-1;p} \end{pmatrix}. \tag{12}$$

The second order empirical moments of individual i make up the $p(p-1)/2 \times 1$ vector s_i, that is

$$\mathrm{s}_i = \begin{pmatrix} (y_{i1} - \mu_1)(y_{i2} - \mu_2) \\ (y_{i1} - \mu_1)(y_{i3} - \mu_3) \\ \vdots \\ (y_{ip-1} - \mu_{p-1})(y_{ip} - \mu_p) \end{pmatrix}. \tag{13}$$

The vector of first and second order empirical deviations for individual i is

$$\mathbf{e}_i = \begin{pmatrix} \text{vec}(\mathbf{1}_{\mathbf{y}_i}) - \text{vec}(\boldsymbol{\nu}) \\ \text{s}_i - \boldsymbol{\sigma} \end{pmatrix}. \tag{14}$$

The size of $\mathbf{e}_i$ is $[p(l-1) + p(p-1)/2] \times 1$ which we refer to as $p^* \times 1$ in the following. An ordinal factor model that does not assume a specific model structure is referred to as a saturated model. The $p^* \times 1$ parameter vector of the saturated model is $\beta = (\text{vec}(\boldsymbol{\nu}), \boldsymbol{\sigma})'$. Equations 5 to 9 show that β is a function of θ. The parameters of the structured model θ can therefore be estimated through minimization of an objective function, that is

$$F_{GEE}(\theta) = \sum_{i=1}^{n} \mathbf{e}_i' \, \mathbf{W}^{-\mathbf{1}} \mathbf{e}_i. \tag{15}$$

This GEE fitting function minimizes the deviations of the individual empirical first and second order moments from the saturated model parameters.

The weight matrix $\mathbf{W}$ is defined as the working covariance matrix of $\mathbf{1}_{\mathbf{y}_i}$ and s_i. From Reboussin and Liang (1998), we derive that a choice for this matrix, that is adequate for the case of non-binary observed variables, is

$$\mathbf{W} = \begin{pmatrix} \mathbf{W_1} & \mathbf{0} \\ \mathbf{0} & \mathbf{W_2} \end{pmatrix}. \tag{16}$$

$\mathbf{W_1}$ is the working covariance matrix of $\mathbf{1}_{\boldsymbol{y_i}}$ $\forall\, i = 1, \ldots, n$, that is

$$\begin{aligned} [\mathbf{W_1}]_{jksh} &= \mu_j(1-\mu_j) && \text{if } \; j = s, k = h, \\ & P(Y_j > k) - \nu_{jk}\nu_{jh} = \Phi(-\tau_{jk}) - \nu_{jk}\nu_{jh} && \text{if } \; j = s, k > h, \\ & P(Y_j > k, Y_s > h) - \nu_{jk}\nu_{sh} = \Phi_2(-\tau_{jk}, -\tau_{sh}, \sigma^*_{js}) - \nu_{jk}\nu_{sh} && \text{if } \; j \neq s, k \neq h. \end{aligned} \tag{17}$$

$\mathbf{W_2}$ is the diagonal working covariance matrix of $\text{s}_{\boldsymbol{i}}$ $\forall\, i = 1, \ldots, n$, with all non-diagonal elements equal to zero and all diagonal elements equal to

$$[\mathbf{W_2}]_{js,js} = \frac{\sum_{i=1}^{n} (\text{w}_{ijs}^2)}{n} - \sigma_{js}^2. \tag{18}$$

Let Δ be the first derivative of β with respect to θ, that is

$$\Delta = \frac{\partial \beta(\theta)}{\partial \theta} = \begin{pmatrix} \frac{\partial \text{vec}(\boldsymbol{\nu})(\theta)}{\partial \theta} \\ \frac{\partial \boldsymbol{\sigma}(\theta)}{\partial \theta} \end{pmatrix}. \tag{19}$$

Then, the first derivative of Equation 15 with respect to θ is

$$\frac{\partial F_{GEE}(\theta)}{\partial \theta} = \frac{\partial F_{GEE}(\beta)}{\partial \beta} \frac{\partial \beta(\theta)}{\partial \theta} = \sum_{i=1}^{n} -2\mathbf{e}_i' \mathbf{W}^{-1} \Delta. \tag{20}$$

Transposing the set of $1 \times p$ row vectors resulting from Equation 20 leads to the following set of estimating equations:

$$\sum_{i=1}^{n} \psi(\boldsymbol{y}_i, \theta) = \sum_{i=1}^{n} \Delta' \mathbf{W}^{-1} \mathbf{e}_i = \mathbf{0}. \tag{21}$$

The model parameters in θ are estimated by solving this set of quadratic estimating equations for θ by iteratively updating the estimator via a modified Fisher's scoring algorithm

$$\hat{\theta}^{r+1} = \hat{\theta}^{r} + (n \cdot \Delta' \mathbf{W}^{-1} \Delta)^{-1} \sum_{i=1}^{n} \psi(\boldsymbol{y}_i, \hat{\theta}), \tag{22}$$

where $\hat{\theta}^r$ denotes the parameter estimates at the rth iteration.
Equation 21 is the *score function* (see Stefanski & Boos, 2002) of the GEE estimation method that can be used for the score-based parameter instability test.

Relation of GEE and WLS Model Scores

In order to estimate model parameters via GEEs, all three components of Equation 21 need to be updated step by step. This means that Equation 5 to 22 need to be computed at every iteration. In contrast to this, the WLS estimation method introduced by Muthén (1983, 1984) works without iteratively updating $\mathbf{W}$, Δ, or $\mathbf{e}_i$. In the first estimation step in the model fitting process, the first and second order sample statistics are estimated following the approach established by Olsson (1979). These sample statistics consist of the sample thresholds t_{jk}, and the bivariate polychoric

	Type I error rate		Power (only $\boldsymbol{\lambda}$-fluctuation)	
	GEE	WLS	GEE	WLS
DM	0.03	0.05	0.32	0.68
CvM	0.03	0.05	0.43	0.60
maxLM	0.03	0.04	0.40	0.63
maxLMo	0.04	0.05	0.42	0.60
WDMo	0.06	0.06	0.32	0.71
LMuo	0.02	0.05	0.35	0.66

Table 1
Power and Type I error rate of score-based test for a multidimemensional GRM model with 9 *dichotomous observed variables and* 3 *latent variables. Comparison of GEE scores vs. WLS scores.*

correlations ρ_{js} for all $k = 1, \ldots, l$, $j, s = 1, \ldots, p$ when $j \neq s$. They make up the $p^* \times 1$ vector $\hat{\boldsymbol{\kappa}}$.

In the third estimation step, the model parameters in θ are estimated through minimization of the objective function

$$F_{OFA}(\theta) = [\hat{\boldsymbol{\kappa}} - \boldsymbol{\kappa}(\theta)]' \, \mathbf{W}^{-1} \, [\hat{\boldsymbol{\kappa}} - \boldsymbol{\kappa}(\theta)]. \tag{23}$$

Note the distinction between the thresholds that are estimated as sample statistics in the first two steps of the model fitting process and the threshold parameters τ_{jk} in θ. Furthermore, $\mathbf{W}$ is a consistent estimator asymptotic covariance matrix of $\hat{\boldsymbol{\kappa}}$ (see Muthén & Satorra, 1995). The weight matrix accounts for multivariate non-normality in the observed variables. This idea goes back to Browne (1984) who focussed primarily on continuous, non-normal observed variables. Muthén (1984) then extended this approach to ordered categorical observed variables. Thus, the WLS estimator is often referred to as an *asymptotically distribution free* estimator (Flora & Curran, 2004; Kyriazos et al., 2018).

In the main text, we show that the application of $\tilde{\psi}(\boldsymbol{y}_i, \hat{\theta})$ to score-based parameter instability tests is computationally efficient and has a low Type I error rate as well as high power. In fact, it outperforms score-based parameter instability tests for models fitted with a full information approach on all metrics.

In Table 1, the performance of the score functions of Equation 21 and the pseudo score function the main text applied to the score-based parameter instability test are compared. To measure the performance of the score-based test for the GEE estimation method, we used the same simulation setup as in the main text but only for a simple multidimemensional GRM model with 3 latent variables and dichotomous observed variables. Also, just one scenario is applied for parameter fluctuation in the data: only the discrimination parameters λ_j differ within a single data set. The results shown in Table 1 indicate that the WLS estimation method outperforms the GEE estimation method with respect to test power.

References

Browne, M. W. (1984). Asymptotically distribution-free methods for the analysis of covariance structures. *British journal of mathematical and statistical psychology*, *37*(1), 62–83.

Flora, D. B., & Curran, P. J. (2004). An empirical evaluation of alternative methods of estimation for confirmatory factor analysis with ordinal data. *Psychological methods*, *9*(4), 466.

Kyriazos, T. A., et al. (2018). Applied psychometrics: sample size and sample power considerations in factor analysis (efa, cfa) and sem in general. *Psychology*, *9*(08), 2207.

Maydeu-Olivares, A. (2005). Linear item response theory, nonlinear item response theory and factor analysis: a unified framework. In A. Maydeu-Olivares & J. J. McArdle (Eds.), *Contemporary psychometrics: A festschrift for roderick p. mcdonald* (pp. 73–102). Lawrence Erlbaum Associates Publishers.

Muthén, B. (1983). Latent variable structural equation modeling with categorical data. *Journal of Econometrics*, *22*(1-2), 43–65.

Muthén, B. (1984). A general structural equation model with dichotomous, ordered categorical, and continuous latent variable indicators. *Psychometrika*, *49*(1), 115–132.

Muthén, B. (1997). Robust inference using weighted least squares and quadratic estimating equations in latent variable modeling with categorical and continuous outcomes. *Psychometrika*.

Muthén, B., & Satorra, A. (1995). Technical aspects of muthén's liscomp approach to estimation of latent variable relations with a comprehensive measurement model. *Psychometrika*, *60*(4), 489–503.

Olsson, U. (1979). Maximum likelihood estimation of the polychoric correlation coefficient. *Psychometrika*, *44*(4), 443–460.

Reboussin, B. A., & Liang, K.-Y. (1998). An estimating equations approach for the liscomp model. *Psychometrika*, *63*, 165–182.

Stefanski, L. A., & Boos, D. D. (2002). The calculus of m-estimation. *The American Statistician*, *56*(1), 29–38.
